# Effect of dental bleaching on pulp oxygen saturation in maxillary central incisors - a randomized clinical trial

**DOI:** 10.1590/1678-7757-2018-0442

**Published:** 2019-04-11

**Authors:** Lorena Ferreira LIMA, Ana Helena Gonçalves de ALENCAR, Daniel de Almeida DECURCIO, Julio Almeida SILVA, Isabella Negro FAVARÃO, Marco Antônio Zaiden LOUREIRO, Fernando Branco BARLETTA, Carlos ESTRELA

**Affiliations:** 1 Universidade Federal de Goiás, Faculdade de Odontologia, Departamento de Ciências Estomatológicas, Goiânia, Goiás, Brazil.; 2 Universidade Luterana do Brasil, Faculdade de Odontologia, Departamento de Ciências Estomatológicas, Canoas, Rio Grande do Sul, Brazil.

**Keywords:** Dental bleaching, Dental pulp, Clinical trial, Tooth bleaching

## Abstract

**Objective:**

To assess pulp oxygen saturation levels (SaO_2_) in maxillary central incisors after dental bleaching.

**Materials and Methods:**

80 participants (160 teeth) were randomly allocated to four groups: G1 In-office bleaching with two applications of 35% hydrogen peroxide (HP) (20 minutes), followed by at-home bleaching with 10% carbamide peroxide (CP) (2 hours/day for 16 days); G2 - Same protocol as G1, plus desensitizing toothpaste; G3 - In-office bleaching with 35% HP and one application of placebo gel (20 minutes), followed by at-home bleaching with 10% CP (2 hours/day for 16 days); and G4 - Same protocol as G3, plus desensitizing toothpaste. Pulp SaO_2_ levels were measured before (T0) and immediately after (T1) in-office bleaching; on the 5^th^ (T2), 8^th^ (T3), 12^th^ (T4), and 16^th^ days of at-home bleaching (T5); and on the 7^th^ (T6) and 30^th^ (T7) days. Mean (SD) pulp SaO_2_ levels were compared within groups by generalized estimating equations (GEE) and Student’s t-test (P<0.05).

**Results:**

Mean pulp SaO_2_ at T0 was 84.29% in G1, 84.38% in G2, 84.79% in G3, and 85.83% in G4. At T1, these values decreased to 81.96%, 82.06%, 82.19%, and 81.15% in G1, G2, G3, and G4 respectively, with significant difference in G4 (P<0.05). During home bleaching, pulp SaO_2_ levels varied in all groups, with 86.55%, 86.60%, 85.71%, and 87.15% means at T7 for G1, G2, G3, and G4, respectively; G2 presented significant difference (P<0.05).

**Conclusions:**

Pulp SaO_2_ level in maxillary central incisors was similar at baseline, reducing immediately after in-office bleaching, regardless of using desensitizing toothpaste and increasing at 30 days after dental bleaching.

## Introduction

The search for a smile that conveys health and beauty is a common reason for seeking dental care. Interpersonal relationships and self-esteem also seem to be associated with the search for dental bleaching. The preference for whitening is justified because it is considered a conservative, safe, and effective treatment.^1^

There are two types of dentist-supervised bleaching techniques: at-home or in-office bleaching,^2^ and both techniques usually employ products based on hydrogen peroxide or carbamide peroxide.^3^ However, some authors have proposed a combined bleaching technique^1^ to combine the benefits of both techniques, such as the minimal adverse effects of at-home bleaching^4^ and the faster whitening potential of in-office bleaching.^2^

Despite the aesthetic benefits, tooth sensitivity (TS) is a very common side effect^5^ of dental bleaching, causing discomfort in two-thirds of patients who undergo dental bleaching.^6^ A recent multivariable logistic regression analysis reported a 51% probability of developing TS after home bleaching and 62.9% after in-office bleaching.^1^ Given this context, some authors have proposed changes in bleaching techniques, such as the reduction of contact time of bleaching gels in in-office procedures,^7^ ^,^ ^8^ the addition of substances to bleaching agents, and the use of desensitizing agents or dentifrices.^3^ ^,^ ^9^

Studies have discussed the penetration of bleaching agents into the pulp chamber.^10^ ^,^ ^11^ Hydrogen peroxide and its products have the ability to rapidly diffuse through the mineralized tissues of the tooth, which is attributed to the low molecular weight of these substances and to the permeability of enamel and dentin.^10^ ^,^ ^11^ The volume of bleaching agent that enters the pulp chamber depends on the peroxide concentration,^8^ ^,^ ^10^ the duration of contact with the tooth structure,^8^ ^,^ ^12^ the enamel and dentin thickness of whitened teeth,^13^ and the presence of restorations.^11^

Hydrogen peroxide after penetrating the pulp chamber comes into contact with the dental pulp, crosses cell membranes and dissociates into free radicals in the cytoplasm, resulting in the establishment of an oxidative stress state.^8^ ^,^ ^14^ The adverse effects of oxidative stress correlate negatively with the enamel/dentin thickness of the whitened tooth. Therefore, small teeth such as mandibular incisors are more susceptible to oxidative damage, being more amenable to diffusion of the bleaching agent into the pulp chamber.^13^ A histopathological study demonstrated pulpitis and superficial necrosis in incisors of young patients after in-office bleaching. In premolars, changes were similar to those observed in unbleached teeth.^13^ High macrophage density, collagen degradation, and inflammatory infiltration of the dental pulp were observed in molars 7 days after completion of in-office bleaching procedures.^15^

The clinical diagnosis of pulp status remains highly challenging. Due to being located within a closed cavity, the dental pulp is inaccessible to direct inspection. Thermal, electrical, and cavity tests are usually employed to assess its clinical condition. The limitations of these pulp sensitivity tests include the possibility of false-positive and false-negative results.^16^

Various electronic modalities have assessed the utility of determining pulp status, including spectrophotometry, laser Doppler flowmetry, and pulse oximetry.^16^ ^,^ ^17^ Pulse oximetry has been widely used in medical practice. In dentistry, it is considered a promising resource for measuring oxygen saturation levels (SaO_2_) in dental pulps.^16^ ^,^ ^17^

Oxygen is carried in the body bound to hemoglobin, an iron-containing protein present in red blood cells. Each molecule of hemoglobin can carry up to four molecules of oxygen, this state is described as “saturated” with oxygen (100%). A healthy individual, with healthy lungs, breathing ambient air, shows arterial SaO_2_ levels between 95% and 100%.^19^ In the teeth, the mean values of SaO_2_ recorded by pulse oximetry have ranged from 75%^19^ to 92.60%^20^ in healthy pulp and below 74.6% in necrotic pulp.^21^ These levels seem to vary according to tooth and age group.^22^ In teeth with inflamed or necrotic pulp, the behavior of this parameter remains unclear.^17^ ^,^ ^21^ ^,^ ^23^

Pulse oximetry seems to be an innovative technology for endodontic diagnosis, which would enable the analysis of pulp status during operative dentistry and real-time monitoring of pulp vitality. The measurement of SaO_2_ levels in pulp tissue may provide new perspectives for more precise diagnoses of pulp status, which would certainly prevent unnecessary endodontic interventions and provide an evidence base for additional clinical studies. Given this context, this study aimed to assess SaO_2_ levels in the pulp of healthy human maxillary central incisors and their response to combined dental bleaching.

## Materials and methods

This study was approved by the Research Ethics Committee of Universidade Federal de Goiás, Brazil (pr.# 52047115.2.0000.5083), and was conducted from February to June 2016. The sample size was calculated in WINPEPI. Considering a 95% confidence level, a 5.0 standard deviation, and a 1.0 margin of error, the minimum sample size was estimated at 99 teeth (i.e., 25 teeth per group). Considering two maxillary central incisors per participant, the exclusion of 20% of participants due to inclusion and exclusion criteria, and a 20% attrition rate during follow-up, an initial sample of 100 individuals was defined. The patients included in the study were asked to provide written informed consent required for studies on human beings.

### Study design

This was a randomized, triple-blind (researchers, participants, and outcome assessors) clinical trial with equal allocation. The experimental design followed the CONSORT guidelines.

### Participant selection and inclusion and exclusion criteria

Clinical evaluation was performed in 325 individuals recruited for dental whitening. Of these, 108 individuals met the eligibility criteria, and underwent a clinical examination. After an interview and history evaluation, the patients underwent an intraoral examination that included inspection, palpation, percussion, and evaluation of periodontal health (absence of mobility, recession, and periodontal attachment loss). Cold thermal pulp testing was performed with the Green Endo Ice refrigerant (-26.2°C, Hygenic, Ohio, USA) under cotton-roll insulation. To evaluate pulp sensitivity, the time to patient response in seconds was recorded using a digital timer, and the sensitive stimulus (pain) was recorded on an analogue scale from 0 to 10, with 0 being no pain and 10 representing severe pain. The test was considered negative if there was no response after two 15-second applications of the refrigerant gas, with a 2-minute interval between applications. Periapical radiography was performed to assist in the diagnosis.

Of the 108 subjects examined, five considered the duration of the experiment overlong, one reported hypersensitivity to the cold thermal test, and 14 did not meet the inclusion criteria. The inclusion criteria were ages between 18 to 39 years, healthy maxillary central incisors, a normal periodontal ligament space, no pulp stones or obliterations, no root resorption or fracture, good periodontal health, and complete root formation. Patients who had previously undergone dental bleaching or whitening, smokers, pregnant women, those with a history of cardiovascular disease, those currently on any systemic medication or drugs, those with a history of occlusal or dental trauma, and those with negative pulp tests were excluded.

### Random allocation and blinding

A computerized sealed-envelope randomization method was used by an investigator who was not involved in the clinical procedures to allocate the 80 selected participants across four groups. These groups were coded G1, G2, G3, and G4. Patients were not informed of their group allocation. Codes were placed into opaque white envelopes, which were sealed and stored with each participant’s clinical record. Unblinding was performed at the end of the study.

All clinical interventions were performed by the same practitioner, a specialist in restorative dentistry who was blinded to group allocation. All materials for the bleaching procedure were prepared by another researcher so the intervention product and placebo were indistinguishable. Measurement of pulp oxygen saturation level before, during, and after bleaching procedures was performed by another blinded researcher (a endodontics specialist).

### Clinical intervention

One week before starting the bleaching procedure, participants were advised on oral hygiene and dietary habits. During this visit, impressions of both dental arches were obtained with type II alginate (Plastalgin, Zhermack, NJ, USA), disinfected, and cast with type III dental stone (Asfer Indústria, SP, Brazil). These casts were then used to fabricate 1 mm-thick silicone whitening trays in a vacuum-forming machine (Essence Dental VH, SP, Brazil). During this visit, participants also received an unidentified tube containing toothpaste (Sorriso Fresh, Colgate Palmolive, SP, Brazil) with or without desensitizer (8% arginine and calcium carbonate), depending on their group allocation. They were then instructed to use only this toothpaste whenever brushing. Participants were also advised to manually rub the toothpaste onto all teeth for 1 minute and rinse with plenty of water before applying the trays containing home bleaching gel.

In-office bleaching procedures were performed following the manufacturer’s instructions, after polishing with pumice and water. To protect the oral soft tissues, the lip and tongue retractor and dental dam provided with the Total Blanc Office kit (Nova DFL, RJ, Brazil) were used. Hydrogen peroxide gel (35%) was applied onto the vestibular surface of the anterior teeth and premolars, left to act for the time specified for each group, and suctioned. Teeth were then rinsed with water and dried with cotton.

The following bleaching procedures were performed: G1 – Single-visit in-office bleaching with two applications of 35% hydrogen peroxide (Total Blanc Office, Nova DFL, RJ, Brazil) for 20 minutes each, followed by at-home bleaching with 10% carbamide peroxide (Total Blanc Home, Nova DFL, RJ, Brazil), applied in an individual tray for 2 hours a day on 16 consecutive days, using toothpaste without desensitizer; G2 – In-office and at-home bleaching with the same protocol used in G1, but instead using toothpaste with a desensitizing agent; G3 – Single-visit in-office bleaching with a single application of 35% hydrogen peroxide (Total Blanc Office, Nova DFL, RJ, Brazil) for 20 minutes and a single application of placebo gel (KY, Johnson & Johnson, NJ, USA) for 20 minutes, followed by at-home bleaching with 10% carbamide peroxide (Total Blanc Home, Nova DFL, RJ, Brazil), applied in an individual tray for 2 hours a day on 16 consecutive days, using toothpaste without desensitizer; and G4 – In-office and at-home bleaching with the same protocol used in G3, but instead using toothpaste with a desensitizing agent.

### Pulp oxygen saturation level

**Figure 1:**
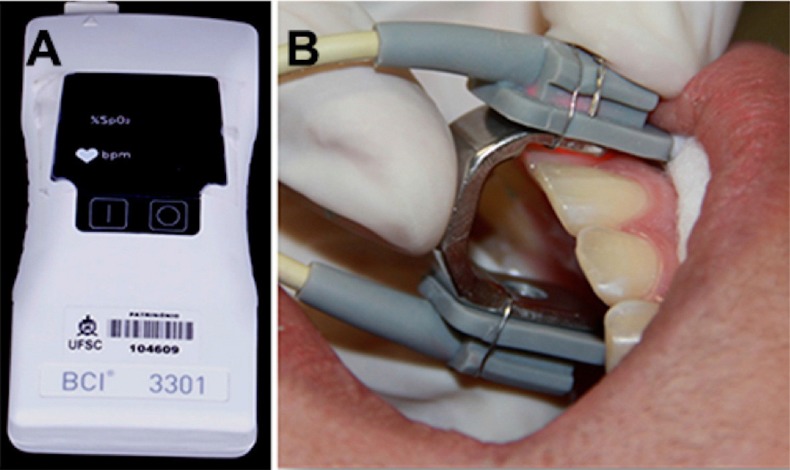
– A) Pulse oximeter and B) sensor with specially manufactured adapter

Fingertip and dental pulp SaO_2_ levels were measured using a BCI portable pediatric pulse oximeter (model 3301, Smiths Medical PM Inc., USA) and a SYS 103 sensor, with a specially made adapter (18 mm high, 18 mm wide, and 11 mm thick), as proposed by Giovanella, et al.^24^ (2014) (Figure 1).

Measurement of pulp SaO_2_ level was performed under cotton-roll isolation and constant suction to ensure dryness of the dental surfaces involved, and in the absence of any reflected light. Participants were placed in the supine position and instructed to remain still during the vitality test. The sensor with the prefabricated adapter was placed onto the tooth of interest so the emitted light reached the middle third of the crown, and the emitting diode and photodetector were coplanar. Two measurements were obtained, one for the first 1 minute after sensor placement onto the tooth, and the second, 1 minute after the first measurement; these measurements were averaged. All measurements were obtained at a controlled room temperature of 24°C (±1°C).

Pulp SaO_2_ was evaluated 1 week before application of the bleaching agent (T0); immediately after the in-office bleaching session (T1); on the fifth day (T2), eighth day (T3), twelfth day (T4), and sixteenth day of home bleaching (T5); and one week (T6) and one month (T7) after the end of all bleaching procedures. Oxygen saturation levels were also evaluated in 10 endodontically treated teeth, which served as negative controls.

### Cold thermal pulp testing

Pulp sensitivity testing was performed during the first visit as described above, and repeated 30 days (T7) after completion of the bleaching procedures, using the same protocol.

### Statistical analysis

Mean fingertip and pulp oxygen saturation levels were described as means and standard deviations. The time points and pulp SaO_2_ levels in each group were compared using the generalized estimating equations (GEE) model. Student’s t*-*test for paired samples was used to compare the overall means at baseline (T0) and 30 days after tooth bleaching (T7). The significance level was set at 5%.

## Results

**Figure 2:**
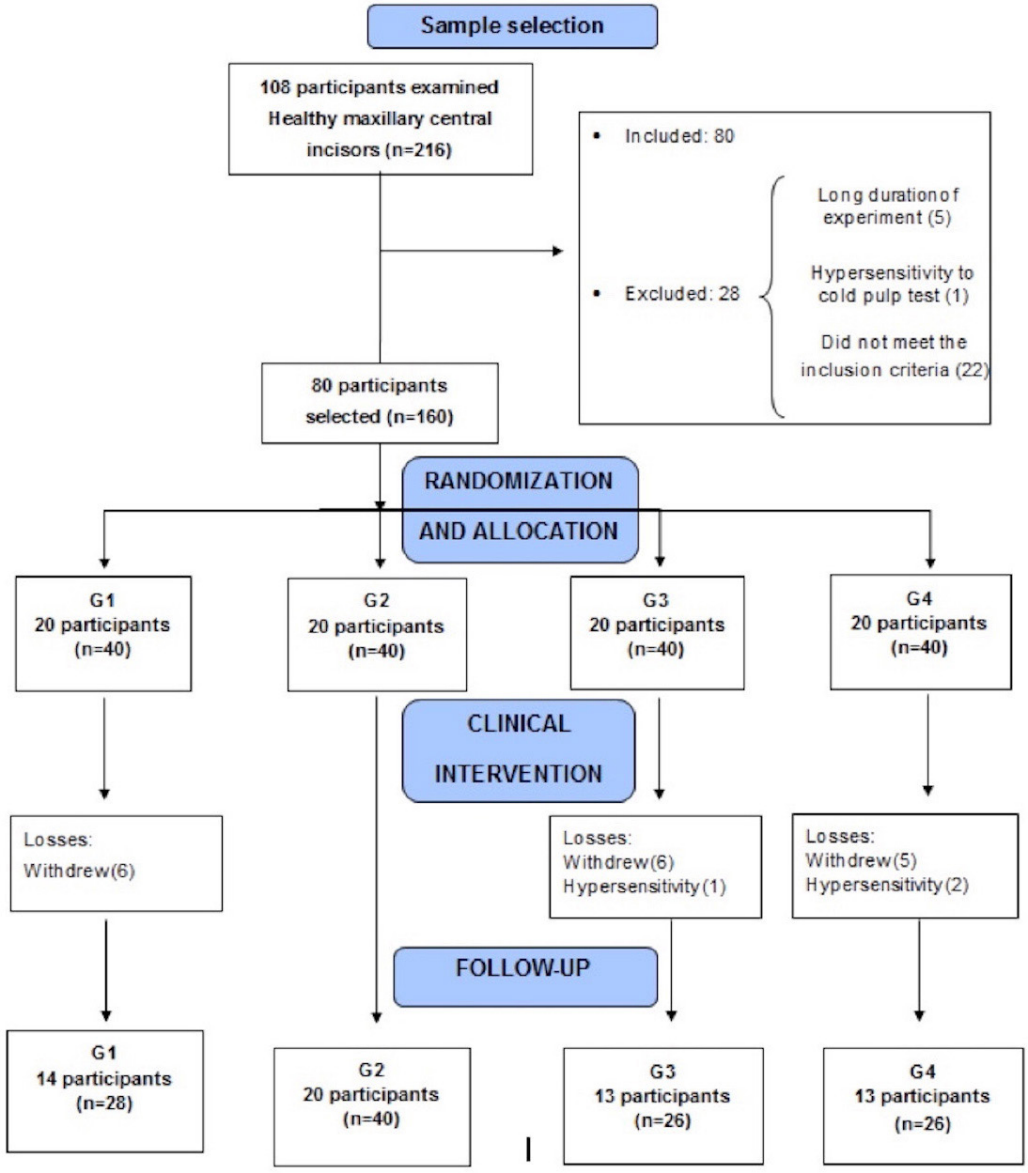
Flow diagram of study inclusion

Of the 80 subjects selected for this study, 21.25% withdrew during the clinical intervention period, and 2.5% were excluded due to severe TS. Pulp SaO_2_ levels were evaluated in 60 subjects (120 maxillary central incisors) before, during, and after combined dental bleaching (Figure 2).

Demographic and clinical characteristics of the subjects are shown in Table 1. Mean pulp oxygen saturation values measured in each group at baseline (T0), immediately after in-office bleaching (T1), during at-home bleaching (T2, T3, T4, and T5), one week (T6), and one month (T7) after the end of the bleaching treatment are presented in Table 2.

**Table 1:** Demographic and clinical characteristics of study participants

Participants	Group 1	Group 2	Group 3	Group 4
	14	20	13	13
Sex (%)				
Women	57.14	75.0	61.54	53.85
Man	42.86	25.0	38.46	46.15
Age group	18-24 years	18-23 years	18-27 years	18-23 years
Systemic disease	0	0	0	0
Mean fingertip oxygen saturation, %	97.48	96.80	97.12	97.34
Healthy maxillary central incisors, n	28	40	26	26
Positive thermal pulp test (cold)	100%	100%	100%	100%

**Table 2:** Mean pulp oxygen saturation and difference (%) at baseline (T0) and during and after dental whitening procedures in each experimental group

Group/Time point			Mean	SD	Difference in means	Occurrence	95% CI of difference in means		P
							Lower limit	Upper limit	
1	T0	T1	81.96	4.47	2.32	Decrease	-0.68	5.33	0.444
		T2	85.39	3.27	-1.11	Increase	-3.58	1.37	0.999
		T3	84.09	4.01	0.20	Decrease	-2.79	3.18	0.999
	Mean 84.29 SD 3.77	T4	83.34	2.70	0.95	Decrease	-2.08	3.97	0.999
		T5	84.57	4.15	-0.29	Increase	-3.87	3.30	0.999
		T6	85.46	3.34	-1.18	Increase	-3.89	1.53	0.999
		T7	86.55	2.94	-2.27	Increase	-4.68	0.14	0.093
2	T0	T1	82.06	4.05	2.31	Decrease	-0.41	5.03	0.222
		T2	84.51	3.51	-0.14	Increase	-2.53	2.25	0.999
		T3	85.20	3.23	-0.83	Increase	-3.04	1.39	0.999
	Mean 84.38 SD 3.56	T4	84.54	2.96	-0.16	Increase	-2.42	2.09	0.999
		T5	84.89	3.23	-0.51	Increase	-2.84	1.81	0.999
		T6	85.31	3.03	-0.94	Increase	-3.21	1.33	0.999
		T7	86.60	2.09	-2.23	Increase	-4.04	-0.41	0.004*
3	T0	T1	82.19	5.17	2.60	Decrease	-1.17	6.36	0.875
		T2	85.00	5.74	-0.21	Increase	-4.47	4.05	0.999
		T3	86.54	4.32	-1.75	Increase	-5.06	1.56	0.999
	Mean 84.79 SD 4.13	T4	84.04	4.19	0.75	Decrease	-1.94	3.44	0.999
		T5	85.94	3.23	-1.15	Increase	-4.21	1.90	0.999
		T6	84.27	2.33	0.52	Decrease	-2.38	3.42	0.999
		T7	85.71	2.53	-0.92	Increase	-3.58	1.74	0.999
4	T0	T1	81.15	3.96	4.67	Decrease	0.72	8.63	0.006*
		T2	84.37	3.81	1.46	Decrease	-2.87	5.79	0.999
		T3	84.38	3.47	1.44	Decrease	-2.54	5.43	0.999
	Mean 85.83 SD 4.56	T4	85.33	2.79	0.50	Decrease	-2.68	3.68	0.999
		T5	86.87	2.77	-1.04	Increase	-3.85	1.77	0.999
		T6	86.85	2.55	-1.02	Increase	-3.95	1.91	0.999
		T7	87.15	1.72	-1.33	Increase	-4.50	1.85	0.999

(SD, standard deviation; 95% CI: 95% confidence interval for difference in means; *Statistically significant difference)

**Figure 3:**
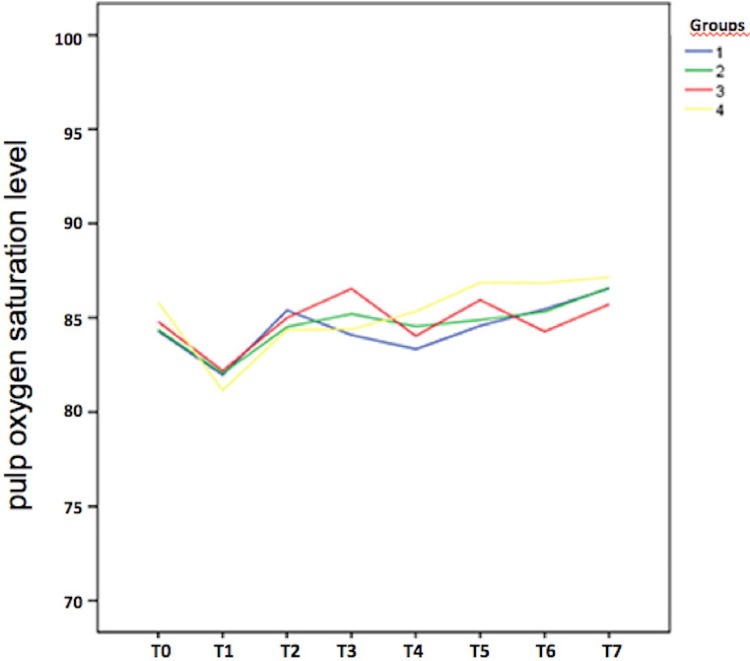
Graphical representation of pulp oxygen saturation level (%) at baseline, variations observed during whitening procedures, and levels at the end of the intervention in Group 1 (blue), Group 2 (green), Group 3 (red), and Group 4 (yellow)

In all groups, the mean pulp SaO_2_ level decreased from T0 to T1, varied somewhat from T1 to T6, and increased from T6 to T7. Table 2 shows that, in G2, there was a significant increase in this value from T0 to T7 (*P*<0.05), while in G4, there was a significant decrease from T0 to T1 (*P*<0.05). Figure 3 illustrates variations in pulp SaO_2_ levels within each group at baseline, during the in-office and at-home bleaching procedures, and after all bleaching treatments had been completed.

The baseline (T0) mean pulp oxygen saturation level in the overall sample (*n*=120) was 84.76%. Thirty days after completion of the bleaching procedures (T7), this level had increased significantly to 86.52% (*P*<0.05). All teeth responded positively to the cold thermal pulp test at T7. None of the 10 endodontically treated teeth (negative controls) had a measurable pulp SaO_2_.

## Discussion

The mean baseline pulp SaO_2_ level in this sample of 120 healthy maxillary central incisors was 84.76%. This value is higher than the 81.25% reported by Stella, et al.^25^ (2015) in an analysis of 110 maxillary central incisors, and similar to the 84.80% reported by Bargrizan, et al.^26^ (2016) in a sample of 190 healthy central incisors. Oxygen saturation values higher than those of this study (92.60%) were measured in maxillary incisors by Kataoka, et al.^18^ (2016). In a critical review of clinical trials using pulse oximetry, Bruno, et al.^23^ (2014) reported an 87.73% average saturation level in 288 maxillary central incisors. Anatomical variations and differences in sample size may justify the heterogeneity of these results.

Studies have reported variability according to different age ranges, such as from 9 to 14 years,^26^ 15 to 40 years,^16^ ^,^ ^19^ ^,^ ^25^ 15 to 55 years,^23^ and 35 to 65 years.^20^ In this study, participants aged 18 to 27 years were selected. The discrepancy in baseline SaO_2_ levels compared to previous studies could also be attributed to these differences in the evaluated age group. Estrela, et al.^22^ (2017) reported lower pulp SaO_2_ levels (80.0%) in healthy premolars of patients aged 40 to 44 years when compared to those measured in patients aged 20 to 24 years (89.71%). This reduction may be justified by the increase in dentin thickness that occurs with aging.^27^

In this study, after an in-office bleaching session (T1) with 35% hydrogen peroxide for 20 minutes, a decrease was observed in pulp SaO_2_ level when compared to baseline (T0): 81.96% in G1, 82.06% in G2, 82.19% in G3, and 81.15% in G4, with a significant difference in G4. Cartagena, et al.^28^ (2015), using laser Doppler flowmetry, reported a reduction in pulp blood flow in the maxillary central incisors immediately after an office bleaching session with application of 35% hydrogen peroxide gel for 15 minutes. This decrease in blood flow may explain the reduction in SaO_2_ levels detected in our study.

Hydrogen peroxide and its products have been shown to rapidly diffuse in teeth with reduced dentin thickness – like the incisors –, causing oxidative stress to the pulp; this damage is proportional to the duration of contact and concentration of the bleaching gel.^8^ ^,^ ^12^ One of the side effects of oxidative stress is the formation of reactive oxygen species (ROS), which cause damage to lipids, nucleic acids, and proteins. Oxidative stress increases when the concentration of ROS exceeds the ability of cells to remove them and repair damage. Depending on the intensity of oxidative stress, the affected cells can suffer membrane ruptures and death. Cell death releases lysosomal enzymes, resulting in extensive tissue damage.^29^ *In vivo*, oxidative stress triggers an inflammatory response.^14^ Benetti, et al.^30^ (2018) observed that IL-6 and IL-17 participated in the inflammatory process occurring in rat pulp tissue after tooth bleaching, particularly in early periods. Immunolabelling was greater with increased H_2_O_2_ concentration and was accompanied by the prolonged activation of CD5-positive cells.

In dental pulp, as in other tissues, inflammation increases blood flow and vascular permeability. However, because the inflammatory process is contained within rigid walls and access to vascularization occurs only through the apical foramen, intrapulpal pressure rises rapidly, with vessel compression and a consequent decrease in pulp blood flow,^31^ ^,^ ^32^ which explains the reduction in SaO_2_ levels detected in this study. According to Alghaithy & Qualtrough^33^ (2017), changes in SaO_2_ during inflammatory processes in the pulp may be related to increased acidity and metabolic rate, which are determinants of hemoglobin deoxygenation.

Studies under hypoxic conditions have been performed with dental pulp cells *in vitro*.^32^ ^,^ ^34^ Ohzeki and Takahashi^35^ (1980) suggested that degenerative changes in the dental pulp were consequences of hypoxia, whereas Wang, et al.^32^ (2010) did not observe any direct damage to pulp cells in this setting. Further clinical studies are needed to evaluate the effects of declining oxygen saturation levels on the dental pulp.

The significant reduction in SaO_2_ level from baseline is particularly noteworthy since this study was performed on healthy, young teeth, with a favorable pulp status and ample perspectives for reoxygenation after restoration of normal blood flow. However, in teeth with restorations there is a possibility of significant enough reductions in pulp oxygen saturation level following bleaching procedures to lead to extensive and irreversible tissue damage.^36^ ^,^ ^37^

Shorter application times and lower bleaching agent concentrations may minimize the toxic effects of hydrogen peroxide on pulp tissue.^12^ In this study, one or two in-office, 20-minute applications of bleaching gel, without prior use of desensitizing toothpaste, did not lead to a significant change in pulp oxygen saturation levels. The reduction in saturation was significant in the group that received desensitizer toothpaste after a single in-office application of bleaching gel. Given the existence of an associaiton between the permeability of exposed dentin and sensitivity, many treatment approaches have endeavored to occlude the oral ends of the dentinal tubules using desensitizing agent in dentifrice formulations.^6^ However, this result showed that the use of desensitizing agents did not prevent penetration of hydrogen peroxide into the pulp which corroborates the findings of Soares, et al.^12^ (2013).

Pulp SaO_2_ levels varied during the home bleaching period (T2 to T5), although higher than at T1. Studies conducted after home bleaching showed that, in contact with the dental surface, carbamide peroxide dissociates and releases only 3.3% of its total hydrogen peroxide concentration. This release occurs slowly and gradually, preventing the immediate penetration of large amounts of hydrogen peroxide into the pulp chamber.^38^ This may explain the variations in pulp oxygen saturation observed in this study during home bleaching, at levels close to those measured at baseline (T0).

On the 30^th^ day after the end of the bleaching sessions (T7), mean pulp SaO_2_ was significantly higher (86.52%) than at baseline (84.76%). Soares, et al.^32^ (2015) demonstrated that pulp cells exposed to bleaching protocols and suffering from reduced oxidative stress were able to proliferate significantly over time, with a three- to fourfold recovery of viability 3 days after bleaching procedures. Cartagena, et al.^28^ (2015), using laser Doppler flowmetry, showed an increase in pulp blood flow to levels above baseline 7 days after the end of bleaching procedures in healthy central incisors.

Physiological blood flow in the dental pulp is approximately 0.4-0.5 mL/min/g, a rate similar to that of the brain and lower than those of the heart or kidney.^31^ When ischemia is transient, pulp viability is generally restored by reoxygenation following increased blood flow. Hypoxia can increase the mitochondrial and proliferative function of pulp cells,^34^ perhaps enough to increase the angiogenic potential of human pulp cells.^39^ The resident stem-cell population of the dental pulp is critical in the regeneration of the dentin-pulp complex.^14^ Vaz, et al.^15^ (2016) reported an increase in the number of blood vessels 7 days after completion of bleaching procedures in molars. Further research into the biological behavior of dental pulp cells under hypoxic conditions is needed.

Considering the challenges involved in the diagnosis of pulp status, pulse oximetry has proven to be an important resource for clinical use, as it is non-invasive, precise, direct, quantitative, and user-friendly.^17^ ^,^ ^23^ Several limitations can lead to changes in oxygen saturation measurements, including systemic blood pressure and drug use; however, a careful patient history analysis and blood pressure measurement can minimize the impact of these variables. Factors such as equipment calibration, ambient temperature, incident light, and patient movement, which also interfere with readings,^28^ can be mitigated by obtaining measurements in temperature-controlled rooms, with the lights off, in duplicate, and for a 30-second period.^24^ Dental morphology aspects involved in variability in measurements can be controlled by stratifying analyses by tooth type and age range. All of these factors were carefully considered in our study. Some factors, however, are beyond the reach of the clinician’s control, such as individual variations in blood flow and neurovascular response, differences in the optical properties of the dental structure, and the presence of surrounding tissues.

In addition, using pulse oximeters for pulp SaO_2_ measurement requires the fabrication of special adapters for fingertip sensors since there are no commercial models specifically produced for teeth, which makes it difficult to keep the emitter and receiver diodes parallel.^23^ ^,^ ^33^ Currently, the distance between the emitting and receiving diodes represents the greatest disadvantage in pulse oximetry equipment because of its substantial influence on the signal-to-noise ratio.^28^ ^,^ ^33^

Oxygen is essential for the aerobic production of cellular energy sources such as adenosine triphosphate (ATP) for mitochondria, and cell activities during tissue repair are directly related to tissue oxygen levels. In the brain, when blood flow decreases to levels under 40% of control values and it is not restored within three minutes, aerobic metabolism causes irreversible tissue damage due to lack of energy.^40^ The dental pulp is surrounded by hard tissue and only has access to vasculature through small openings at the root apex, which makes it susceptible to hypoxia. Decreased pulpal blood flow has been reported to cause severe pulp damage.^41^ According to Ueno, et al.^42^ (2006), when pulp cells were exposed to hypoxia for periods of 24 hours there was interruption of cell growth and death was detected. Under severe hypoxia conditions, the cells seem to not survive, initiating events that lead to cell death by apoptosis.^43^ Therefore, monitoring pulp oxygen saturation levels before, during, and after tooth bleaching procedures is fundamental.

## Conclusion

The pulp SaO_2_ level in maxillary central incisors was similar at baseline, reducing immediately after in-office bleaching, regardless of the use of desensitizing toothpaste and increasing at 30 days after dental bleaching.
